# Dry Swabs and Dried Saliva as Alternative Samples for SARS-CoV-2 Detection in Remote Areas in Lao PDR

**DOI:** 10.1093/ofid/ofae433

**Published:** 2024-07-23

**Authors:** Bountoy Sibounheuang, Latsaniphone Boutthasavong, Danoy Chommanam, Koukeo Phommasone, Siribun Panapruksachat, Viladeth Praphasiri, Sengvong Bouttavong, Hongkham Sisavath, Nathaniel C V Christy, Andrew G Letizia, Mayfong Mayxay, Manivanh Vongsouvath, Elizabeth A Ashley, Audrey Dubot-Pérès

**Affiliations:** Microbiology Laboratory, Lao-Oxford-Mahosot Hospital-Wellcome Trust Research Unit, Mahosot Hospital, Vientiane, Lao PDR; Microbiology Laboratory, Lao-Oxford-Mahosot Hospital-Wellcome Trust Research Unit, Mahosot Hospital, Vientiane, Lao PDR; Microbiology Laboratory, Lao-Oxford-Mahosot Hospital-Wellcome Trust Research Unit, Mahosot Hospital, Vientiane, Lao PDR; Microbiology Laboratory, Lao-Oxford-Mahosot Hospital-Wellcome Trust Research Unit, Mahosot Hospital, Vientiane, Lao PDR; Microbiology Laboratory, Lao-Oxford-Mahosot Hospital-Wellcome Trust Research Unit, Mahosot Hospital, Vientiane, Lao PDR; Xiengkhouang Hospital, Xiengkhouang, Lao PDR; Luang Namtha Hospital, Luang Namtha, Lao PDR; Attapeu Hospital, Attapeu, Lao PDR; Naval Medical Research Unit INDO PACIFIC, Emerging Infectious Disease Department, Singapore, Singapore; Naval Medical Research Unit INDO PACIFIC, Emerging Infectious Disease Department, Singapore, Singapore; Microbiology Laboratory, Lao-Oxford-Mahosot Hospital-Wellcome Trust Research Unit, Mahosot Hospital, Vientiane, Lao PDR; Nuffield Department of Clinical Medicine, Centre for Tropical Medicine and Global Health, University of Oxford, Oxford, UK; Institute of Research and Education Development, University of Health Sciences, Vientiane, Lao PDR; Microbiology Laboratory, Lao-Oxford-Mahosot Hospital-Wellcome Trust Research Unit, Mahosot Hospital, Vientiane, Lao PDR; Microbiology Laboratory, Lao-Oxford-Mahosot Hospital-Wellcome Trust Research Unit, Mahosot Hospital, Vientiane, Lao PDR; Nuffield Department of Clinical Medicine, Centre for Tropical Medicine and Global Health, University of Oxford, Oxford, UK; Microbiology Laboratory, Lao-Oxford-Mahosot Hospital-Wellcome Trust Research Unit, Mahosot Hospital, Vientiane, Lao PDR; Nuffield Department of Clinical Medicine, Centre for Tropical Medicine and Global Health, University of Oxford, Oxford, UK; Unité des Virus Émergents (UVE: Aix-Marseille Univ, Università di Corsica, IRD 190, Inserm 1207, IRBA), Marseille, France

**Keywords:** COVID-19, dry saliva spot, dry swab, resource-limited settings, SARS-CoV-2

## Abstract

**Background:**

Surveillance of SARS-CoV-2 circulation is mainly based on real-time reverse transcription–polymerase chain reaction, which requires laboratory facilities and cold chain for sample transportation. This is difficult to achieve in remote rural areas of resource-limited settings. The use of dried blood spots shipped at room temperature has shown good efficiency for the detection of arboviral RNA. Using a similar approach, we conducted a study at 3 provincial hospitals in Laos to compare the detection of SARS-CoV-2 from neat and dried spot samples.

**Methods:**

Between January 2022 and March 2023, patients with respiratory symptoms were recruited. Nasopharyngeal/oropharyngeal swabs in virus transport medium (VTM), dry swabs, saliva, and dried saliva spotted on filter paper were collected. All samples were tested by SARS-CoV-2 real-time reverse transcription–polymerase chain reaction.

**Results:**

In total, 479 participants were included. The VTM samples tested positive for 288 (60.1%). High positive percent agreements were observed for dry swab (84.8%; 95% CI, 80.2%–88.8%) and saliva (89.2%; 95% CI, 85.1%–92.6%) as compared with VTM. There was a loss of sensitivity when saliva was dried on filter paper (73.6%; 95% CI, 68.1%–78.6%) as compared with saliva. SARS-CoV-2 variant (Delta or Omicron) had no significant impact on the performance of the different sample types.

**Conclusions:**

Our findings suggest that dry swabs could be a good alternative for sample collection and permit easy shipping at ambient temperature for subsequent viral SARS-CoV-2 RNA purification and molecular investigation. This is a useful tool to consider for a rapid implementation of large-scale surveillance of SARS-CoV-2 in remote areas, which could be extrapolated to other respiratory targets during routine surveillance or in the case of a novel emerging pandemic.

Following the declaration by the World Health Organization of the COVID-19 global pandemic on 12 March 2020, strict nonpharmaceutical interventions were rapidly implemented in the Lao PDR (People's Democratic Republic, Laos), including a national lockdown and closure of international borders. After the first SARS-CoV-2–confirmed positive case on 23 March 2020, widespread SARS-CoV-2 transmission in the community was averted until the second half of 2021 [1], with the introduction of the more transmissible Delta and Omicron variants. COVID-19 surveillance with molecular screening was implemented in March 2020 in a few centers in the capital, Vientiane, led by the NCLE (National Center for Laboratory and Epidemiology). Sample testing was performed with reference probe–based real-time reverse transcription–polymerase chain reaction (RT-qPCR) developed in early 2020 by several groups [2, 3] and then commercial assays, following World Health Organization guidelines. The recommendation was to use nasopharyngeal specimens collected in virus transport medium (VTM) as the specimen type showing the highest sensitivity for the detection of respiratory pathogens. The efficacy of other specimens for the detection of SARS-CoV-2 was investigated in many published studies, with saliva samples of particular interest given the ease of collection [4–7]. Good agreement with nasopharyngeal specimens has been observed for the detection of SARS-CoV-2 by RT-qPCR.

In resource-limited settings with a lack of qualified health workers for safe sample collection and processing, as well as limited cold chain for sample shipment to a central laboratory, surveillance of populations in remote areas is difficult to achieve. Given the efficiency documented with saliva samples for the detection of SARS-CoV-2 by RT-qPCR, we hypothesized that saliva could be an alternative specimen to nasopharyngeal/oropharyngeal swabs to support an affordable surveillance program while negating the need for cold chain processes. This is particularly relevant and needs to be evaluated in remote and resource-challenged locations that do not have easy access to supporting reference laboratories, such as provincial areas in Laos. Furthermore, dried blood spot collection and shipment at room temperature have shown good efficiency for arbovirus detection by RT-qPCR in comparison with neat serum [8–10]. In addition, studies showed that SARS-CoV-2 could be amplified by RT-qPCR from dry swabs spiked with positive sample or virus [11–15]. Khan and Roopa recently showed 81% agreement when oropharyngeal dry swab were compared with swabs in VTM collected from 56 patients [16]. We hypothesized that dry nasopharyngeal/oropharyngeal swabs and dry saliva on filter paper shipped at room temperature would be efficient for the detection of SARS-CoV-2 and cheaper than VTM for patient sample collection in remote areas. If those approaches proved efficient, this would greatly facilitate SARS-CoV-2 surveillance in rural areas and permit a better picture of virus circulation throughout the country.

We conducted a study at 3 provincial hospitals in Luang Namtha (LNT), Xiengkhouang (XK), and Attapeu (ATP) and compared the detection of SARS-CoV-2 by RT-qPCR from 2 types of specimens using 2 conditions for sample collection: nasopharyngeal/oropharyngeal swabs in VTM, dry nasopharyngeal/oropharyngeal swabs, saliva, and saliva on filter paper.

## METHODS

### Study Sites

This study was conducted in provincial hospitals at 3 sites in Laos: LNT province, a mountainous region in the far northwest of the country; XK province, in the country's northeast region; and ATP province, in the southeast of the country.

The study was conducted over 2 periods: during the COVID-19 epidemic in early 2022 (January–April) and during a period of low SARS-CoV-2 circulation in early 2023 (February and March).

### Participant Consent Statement

Ethical approval for this study was granted by the Lao National Ethics Committee for Health Research (NECHR 010, 2 March 2021), the Oxford Tropical Research Ethics Committee (OXTREC 53-20, 12 November 2020), and the Naval Medical Research Unit INDO PACIFIC Human Research Protections Officer (HRPO N2.2021.0001), in accordance with all applicable federal regulations governing the protection of human subjects. All participants provided written informed consent.

### Participant Recruitment

Cases were patients with fever and acute respiratory symptoms who presented to provincial hospitals and were recruited as part of a larger ongoing respiratory virus surveillance study. Criteria for inclusion to this substudy were as follows: male or female patients ≥15 years of age who presented with fever (axillary temperature ≥37.5 °C) or history of fever, cough, other respiratory symptoms/signs, or loss of smell/taste and had an onset of illness <10 days and who had given informed consent.

### Specimen Collection

Two pairs of respiratory swabs (nasopharyngeal and oropharyngeal) were collected from all participants: the first collected pair was placed in 1 mL of VTM (VTM sample; Sigma Virocult [MWE]), while the second was placed in a 15-mL sterile conical tube (dry swabs; Falcon [Corning]). VTM was stored at the recruitment site at 2 to 8 °C until aliquoting 2 to 6 hours later into 2 tubes (500 and 200 µL) and then kept at −20 °C.

Around 2 mL of saliva was collected by salivation directly into a 50-mL sterile conical tube (Centristar; [Corning]) and stored at 2 to 8 °C; 2 to 6 hours later, 100 µL of saliva was loaded with a micropipette on a piece of filter paper (3 × 1 cm, 3MM Chr Blotting Paper; Whatman, GE Healthcare Life Sciences), placed inside a 1.5-mL microtube (Eppendorf) containing around 10 silica beads, and left to dry for 2 hours. The rest of the saliva was kept at 2 to 8 °C until shipment to the microbiology laboratory at Mahosot hospital.

All collected samples were shipped once a week in a cool box to the microbiology laboratory at Mahosot hospital, Vientiane, by bus within 24 hours. The temperature was recorded inside the box and checked to be <8 °C on arrival at the laboratory. The dried saliva spots (DSS) and dry swabs were kept at room temperature for 7 days before processing.

### RNA Extraction

VTM samples were immediately extracted by the laboratory technician on duty that day, as part of the routine SARS-CoV-2 surveillance process using the EZ1 Virus Mini Kit (Qiagen) following the manufacturer's instruction from 200 µL of sample with an elution volume of 90 µL.

EZ1 Virus Mini Kit reagents were not available to test the other samples which were tested using the QIAamp Viral Mini Kit (Qiagen), which performed with comparable efficiency (results not shown). Saliva (140 μL) was extracted upon arrival at the laboratory by following the manufacturer's instruction with an elution volume of 80 μL. After extraction, the remaining volume (maximum, 1 mL) was stored at –80 °C. The tips of the dry swabs were cut out and placed into a microtube. ATL buffer (500 μL; Qiagen) was added to the microtube containing the swab tips and to the microtube containing the DSS; after which, both tubes were incubated at 56 °C for 15 minutes. Microtubes were vortexed for 30 seconds before and after incubation. Solution (140 μL) was then submitted to the QIAamp Viral Mini Kit following the manufacturer's instructions with an elution volume of 80 μL.

### RT-qPCR Assay

SARS-CoV-2 was detected by an RT-qPCR system developed by the US Centers for Disease Control and Prevention targeting the N gene [2]. RT-qPCR mix was prepared with the SuperScript III Platinum One-Step qRT-PCR Kit (Invitrogen; Thermo Fisher) by mixing 12.5 μL of 2× Master Mix with 0.5 μL of RT/Taq mix, 7 μL of primer and probe solution (each final concentration of primer [500 nM] and probe [125 nM] via a pre-prepared freeze-dried vial as previously described [17]), and 5 μL of RNA. The RT-qPCR run was performed with the CFX96 Touch Real-Time PCR System (Bio-Rad) with thermal cycling as follows: 50 °C for 15 minutes, 95 °C for 2 minutes, 45× (95 °C for 15 seconds, 60 °C for 45 seconds). The RT-qPCR assays were considered as positive if the quantification cycle (Cq) value was <37.

### SARS-CoV-2 Sequencing for Variant Identification

VTM samples with SARS-CoV-2 RT-qPCR with a Cq value ≤28 were submitted to whole genome sequencing using a MinION Mk1B sequencer equipped with the R9.4.1 flow cell (Oxford Nanopore). The whole genome was amplified using the multiplex tiling reverse transcription–polymerase chain reaction protocol from Oxford Nanopore, based on a protocol by the ARTIC Network. The tiled amplicons were generated using LunaScript RT SuperMix, Q5 HS Master Mix, and Midnight Primers supplied in the Midnight RT PCR Expansion Kit (Oxford Nanopore). Polymerase chain reaction products were then barcoded using Oxford Nanopore Rapid Barcoding Kits (SQK-RBK004 or SQK-RBK110.96) [18]. The sequencing output data were assembled and analyzed with the Nanopore EPI2ME pipeline. The consensus sequences were then classified using Nextclade and Pangolin software (Pangolin version 4.3 and Pangolin-data version 1.22).

### Statistics

RT-qPCR from nasopharyngeal/oropharyngeal swab collected in VTM is an imperfect test for SARS-CoV-2 detection (ie, SARS-CoV-2–positive cases are missed). As such, RT-qPCR results for the different samples were compared: VTM vs saliva, VTM vs dry swabs, VTM vs DSS, and saliva vs DSS. Cohen k coefficient and positive (PPA), negative (NPA), and overall percent agreements were calculated, as recommended by the Food and Drug Administration's guidance on reporting results from studies evaluating diagnostic tests [19] without a gold standard.

## RESULTS

### Participant Inclusion

**Figure 1.**
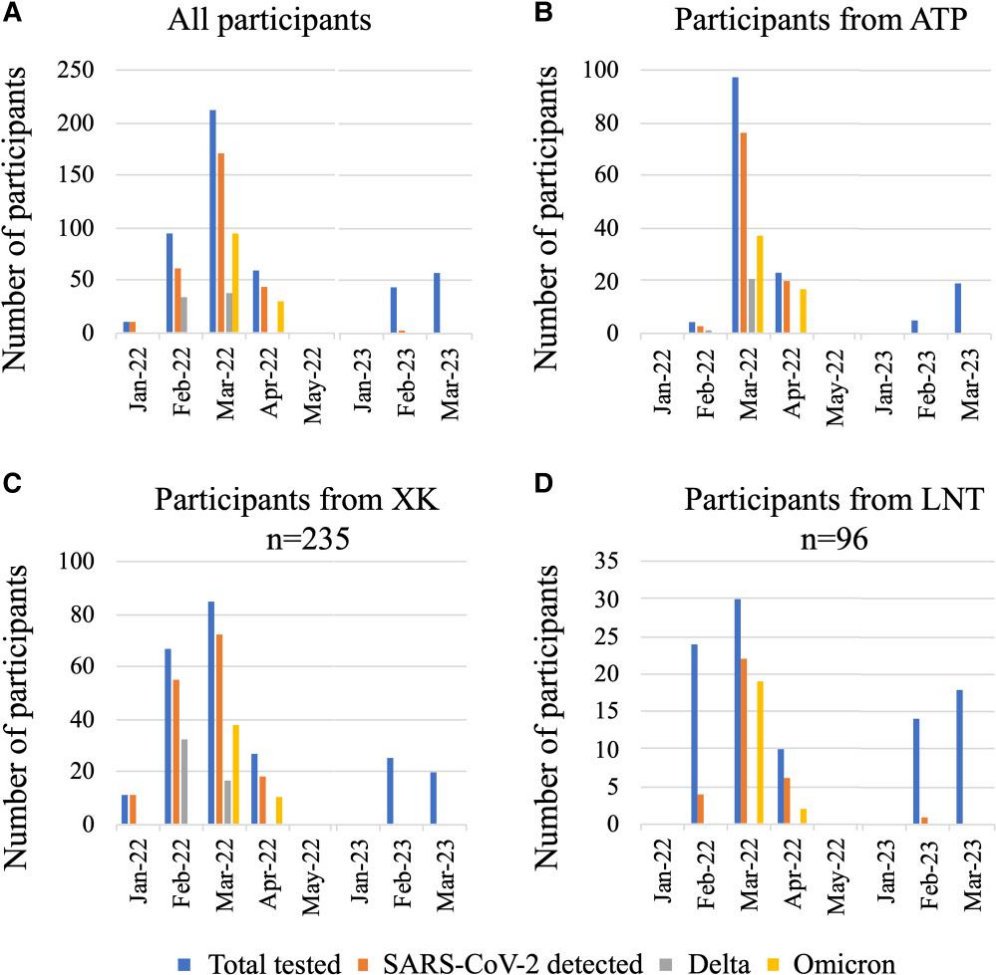
Distribution over time of number of participants in the study, number of participants positive by SARS-CoV-2 real-time reverse transcription-polymerase chain reaction, and number of sequenced Delta and Omicron variants: *A*, for all participants; *B–D*, for participants at the Attapeu (ATP), Xiengkhuang (XK), and Luang Namtha (LNT) sites.

Participants meeting the study inclusion criteria who had all specimens collected were enrolled into the study: 378 in 2022 and 101 in 2023 (N = 479). The participant sex ratio was 0.9 (232:247 male:female), the median age was 35 years (IQR, 27–46), 85.2% reported a sore throat (408/479), and 71.8% had a runny nose (344/479; Supplementary Table 1). The VTM samples tested positive for SARS-CoV-2 by RT-qPCR for 288 of 479 (60.1%) participants: 287 of 378 (75.9%) in 2022 and 1 of 101 in 2023. Participants and COVID-19 case distribution over time and location are displayed in Figure 1.

### Performance of Saliva for SARS-CoV-2 Detection by RT-qPCR

**Figure 2.**
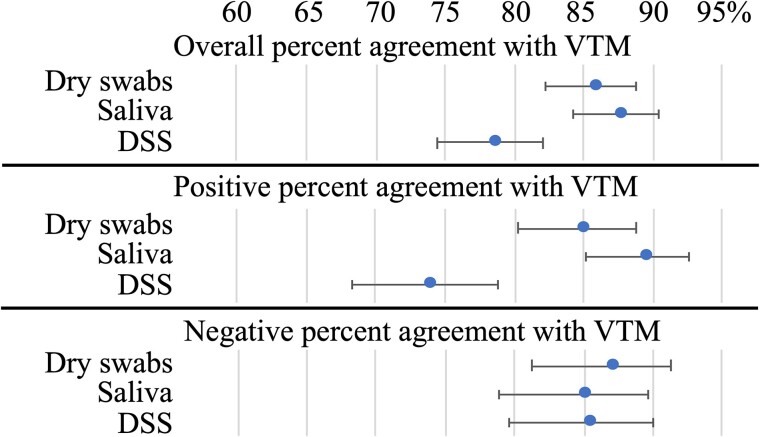
Percent agreements of SARS-CoV-2 real-time reverse transcription-polymerase chain reaction results for dry swabs, saliva, and dried saliva spots (DSS) when compared with results for combined nasopharyngeal/oropharyngeal swabs in virus transport medium (VTM). Error bars indicate 95% CI.

**Figure 3.**
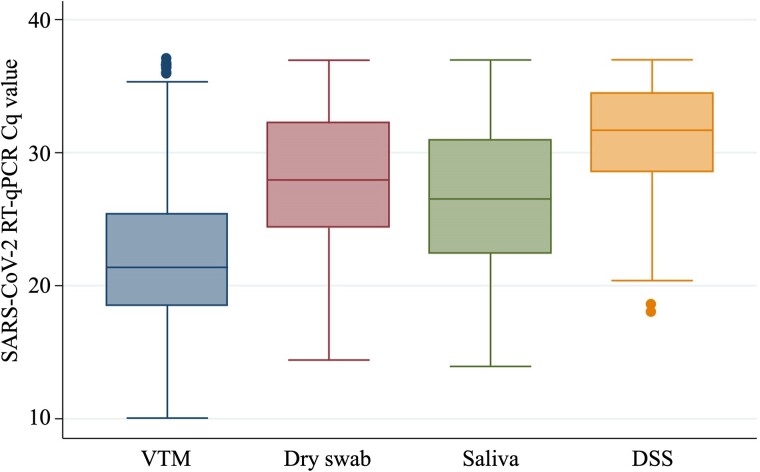
Box plot of SARS-CoV-2 real-time reverse transcription-polymerase chain reaction quantification cycle values, targeting N gene, for combined nasopharyngeal/oropharyngeal swabs in virus transport medium (VTM), dry swab, saliva, and dried saliva spot (DSS) samples. Horizontal line inside the box, median; upper side of the box, 75th percentile; lower side of the box, 25th percentile; horizontal line of the upper bar, upper adjacent value; horizontal line of the lower bar, lower adjacent value; dots at the top or the bottom of the graph, outside values.

Good agreement (Cohen κ coefficient, 0.74) was obtained between SARS-CoV-2 RT-qPCR results from VTM samples and saliva samples (Table 1). Concordant SARS-CoV-2 RT-qPCR results for the 2 sample types occurred for 87.5% of the participants (419/479). The PPA was high (89.2%; Figure 2). However, Cq values from VTM samples were significantly lower than those from saliva samples (Figure 3, Supplementary Figure 1*A*; *t* test, *P* < .001).

**Table 1.** SARS-CoV-2 RT-qPCR Results for VTM and Saliva Samples

	VTM^a^		
	Positive	Negative	Total
Saliva			
Positive	257	29	286
Negative	31	162	193
Total	288	191	479
Percent agreements (95% CI)			
Overall	87.5 (84.2–90.3)		
Positive	89.2 (85.1–92.6)		
Negative	84.8 (78.9–89.6)		
Cohen κ coefficient (95% CI)	0.74 (0.68–0.80)		

Abbreviations: RT-qPCR, real-time reverse transcription–polymerase chain reaction; VTM, virus transport medium.

^a^Combined nasopharyngeal/oropharyngeal swabs in VTM samples.

The PPA was 96.3% when selecting participant with VTM SARS-CoV-2 RT-qPCR Cq <20, 90.7% when selecting 20 ≤ Cq < 30, and 64.1% when selecting 30 ≤ Cq < 37. The NPA was significantly lower in ATP (75.5%; 95% CI, 61%–86.7%) than in LNT (92.1%; 95% CI, 82.4%–97.4%; Supplementary Table 2).

### Performance of the Dry Swabs for SARS-CoV-2 Detection by RT-qPCR

A Cohen κ coefficient of 0.71 was obtained between SARS-CoV-2 RT-qPCR results from VTM samples and dry swab samples (Table 2). The same SARS-CoV-2 RT-qPCR results for the 2 samples occurred for 85.8% of the participants (411/479). The PPA was high (85.1%; Figure 1). However, Cq values from VTM samples were significantly lower than those from dry swab samples (Figure 3, Supplementary Figure 1*B*; *t* test, *P* < .001).

**Table 2.** SARS-CoV-2 RT-qPCR Results for VTM and Dry Swab Samples

	VTM^a^		
	Positive	Negative	Total
Dry swab			
Positive	245	25	270
Negative	43	166	209
Total	288	191	479
Percent agreements (95% CI)			
Overall	85.4 (82.0–88.5)		
Positive	85.1 (80.4–89.0)		
Negative	86.9 (81.3–91.3)		
Cohen κ coefficient (95% CI)	0.71 (0.65–0.77)		

Abbreviations: RT-qPCR, real-time reverse transcription–polymerase chain reaction; VTM, virus transport medium.

^a^Combined nasopharyngeal/oropharyngeal swabs in VTM samples.

The PPA was 93.6% when selecting participants with VTM SARS-CoV-2 RT-qPCR Cq <20, 90.0% when selecting 20 ≤ Cq < 30, and 43.6% when selecing 30 ≤ Cq < 37.

### Performance of DSS for SARS-CoV-2 Detection by RT-qPCR

Comparisons of SARS-CoV-2 RT-qPCR results for DSS samples vs saliva or for DSS vs VTM samples are displayed in Table 3. A Cohen κ coefficient of 0.56 was observed for DSS vs saliva and 0.57 for DSS vs VTM. The PPA was 78% for both comparisons (78.3% and 78.7%; Figure 1). Cq value from DSS sample was higher than Cq value from saliva sample or VTM sample fort 92.5% (196/212 and 198/214, respectively) of the participants tested positive for both of the compared samples (Supplementary Figure 1*C* and 1*D*).

**Table 3.** SARS-CoV-2 RT-qPCR Results for DSS and Saliva or VTM Samples

	Saliva		VTM^a^		
	Positive	Negative	Positive	Negative	Total
DSS					
Positive	212	30	214	28	242
Negative	74	163	74	163	237
Total	286	193	288	191	479
Percent agreements (95% CI)					
Overall	78.3 (74.3–81.9)		78.7 (74.8–82.3)		
Positive	74.1 (68.6–79.1)		74.3 (68.9–79.3)		
Negative	84.5 (78.6–89.3)		85.3 (79.5–90.0)		
Cohen κ coefficient (95% CI)	0.57 (0.49–0.64)		0.57 (0.50–0.65)		

Abbreviations: DSS, dried saliva spots; RT-qPCR, real-time reverse transcription–polymerase chain reaction; VTM, virus transport medium.

^a^Combined nasopharyngeal/oropharyngeal swabs in VTM samples.

The PPA was 86.2% when selecting participants with VTM SARS-CoV-2 RT-qPCR Cq <20, 72.9% when selecting 20 ≤ Cq < 30, and 46.2% when selecting 30 ≤ Cq < 37.

### Impact of the Number of Days of Illness at Sample Collection

Slightly better agreements were observed for all sample comparisons (VTM vs saliva, VTM vs dry swabs, VTM vs DSS, and saliva vs DSS) for participants with <5 days of illness vs 5 to 9 days of illness (Supplementary Table 3).

### Impact of SARS-CoV-2 Variants

SARS-CoV-2 sequences were obtained from 194 of the 237 VTM-positive samples with a RT-qPCR Cq value ≤28. Seventy-one samples collected in February and March 2022 were characterized as the Delta variant and 123 collected in March and April 2022 as the Omicron variant (Figure 1). Seventy-six (51.4%) strains from Attapeu were sequenced: 22 (28.9%) were Delta and 54 (71.0%) were Omicron. Ninety-seven (41.3%) strains from XK were sequenced: 49 (50.5%) were Delta and 48 (49.5%) were Omicron. Twenty-one (21.9%) strains were sequenced from LNT; all were the Omicron variant. GenBank and GISAID accession numbers are provided in supplemental data (Supplementary Table 4).

**Figure 4.**
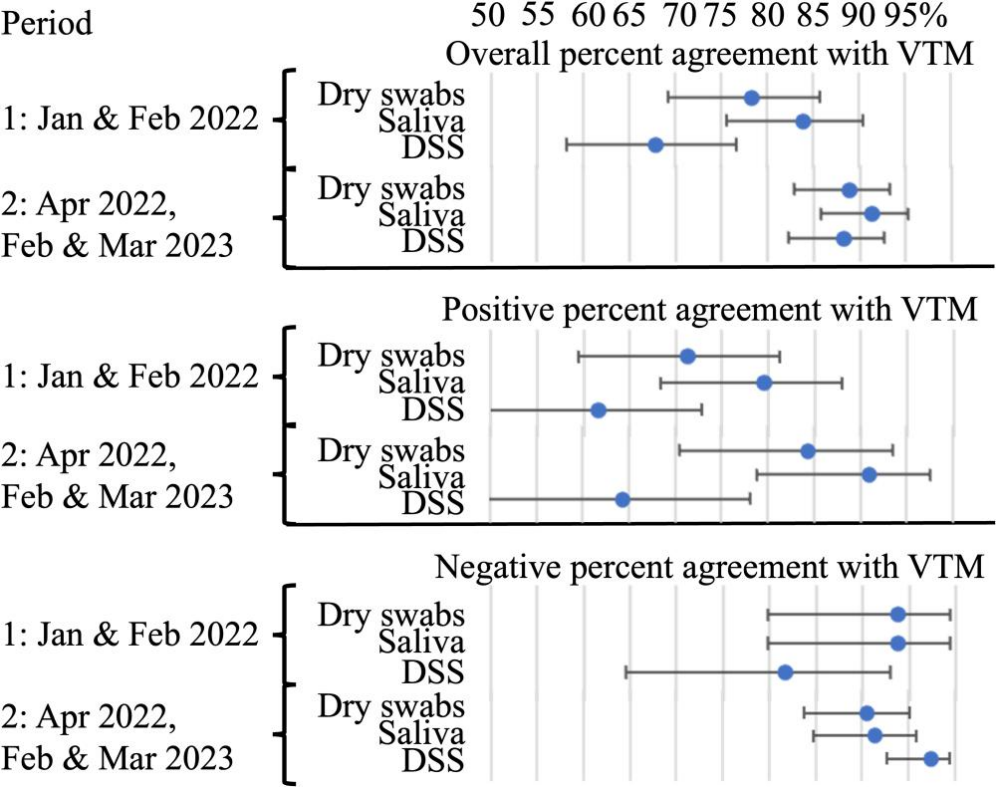
Percent agreements of SARS-CoV-2 real-time reverse transcription-polymerase chain reaction results for dry swabs, saliva, and dried saliva spots (DSS) when compared with results for nasopharyngeal/oropharyngeal swabs in VTM samples for the 2 periods with Delta or Omicron as the main circulating strain. Period 1: January and February 2022, assuming that the main strain circulating was Delta. Period 2: April 2022 and February and March 2023, assuming that the main strain circulating was Omicron. Error bars indicate 95% CI.

The samples submitted to sequencing were based on a Cq value ≤28. Consequently, they are not representative of all positive samples. Therefore, to assess if the detection of SARS-CoV-2 from the different samples could be influenced by the variant, we compared results for 2 periods (Table 4, Figure 4): January and February 2022 (period 1) assuming that the main strain circulating was Delta and April 2022 and February and March 2023 (period 2) assuming that the main strain circulating was Omicron. Percent agreements and κ coefficients were higher in period 2 than in period 1. However, the differences were not statistically significant, and the Cq values for the different samples were not significantly different between period 1 and period 2 (Supplementary Figure 2). The only significant difference was observed for NPA for DSS SARS-CoV-2 RT-qPCR results in comparison with VTM, which were higher during Omicron circulation (period 2) vs Delta circulation (period 1). Yet, period 2 was later in the pandemic, with only 45 of 161 (28.0%) participants positive in comparison with 73 of 106 (68.9%) in period 1. We expected to have a higher NPA during periods of low circulation of the virus.

**Table 4.** SARS-CoV-2 RT-qPCR Results for VTM and Saliva, Dry Swab, or DSS Samples for the 2 Periods With Delta or Omicron as the Main Circulating Strain

	VTM^a^					
	Period 1: Jan + Feb 2022			Period 2: Apr 2022, Feb + Mar 2023		
	Positive	Negative	Total	Positive	Negative	Total
Saliva						
Positive	58	2	60	41	10	51
Negative	15	31	46	4	106	110
Total	73	33	106	45	116	161
Percent agreements (95% CI)						
Overall	84.0 (75.6–90.4)			91.3 (85.8–95.2)		
Positive	79.5 (68.4–88.0)			91.1 (78.8–97.5)		
Negative	93.9 (79.8–99.3)			91.4 (84.7–95.8)		
Cohen κ coefficient (95% CI)	0.66 (0.52–0.80)			0.79 (0.69–0.90)		
Dry swab						
Positive	52	2	54	38	11	49
Negative	21	31	52	7	105	112
Total	73	33	106	45	116	161
Percent agreements (95% CI)						
Overall	78.3 (69.2–85.7)			88.8 (82.9–93.2)		
Positive	71.2 (59.4–81.2)			84.4 (70.5–93.5)		
Negative	93.9 (79.8–99.3)			90.5 (83.7–95.2)		
Cohen κ coefficient (95% CI)	0.56 (0.42–0.71)			0.73 (0.61–0.85)		
DSS						
Positive	45	6	51	29	3	32
Negative	28	27	55	16	113	129
Total	73	33	106	45	116	161
Percent agreements (95% CI)						
Overall	67.9 (58.2–76.7)			88.2 (82.2–92.7)		
Positive	61.6 (49.5–72.8)			64.4 (48.8–78.1)		
Negative	81.8 (64.5–93.0)			97.4 (92.6–99.5)		
Cohen κ coefficient (95% CI)	0.37 (0.21–0.53)			0 .68 (0.55–0.81)		

Period 1: January and February 2022, assuming that the main strain circulating was Delta. Period 2: April 2022 and February and March 2023, assuming that the main strain circulating was Omicron.

Abbreviations: DSS, dried saliva spots; RT-qPCR, real-time reverse transcription–polymerase chain reaction; VTM, virus transport medium.

^a^Combined nasopharyngeal/oropharyngeal swabs in VTM samples.

## DISCUSSION

In line with previous studies, we observed good agreement between saliva and combined nasopharyngeal/oropharyngeal swab samples in VTM for the detection of SARS-CoV-2 by RT-qPCR. We also showed that dry swabs provided good performance for SARS-CoV-2 detection. There was a loss of sensitivity when saliva samples were dried on filter paper. We did not observe a significant impact of the variants (Delta or Omicron) on the performance of the different types of samples for the detection of SARS-CoV-2 by RT-qPCR, although the PPA tended to be higher while Omicron was circulating.

In a pandemic context, with the requirement for mass testing and necessity for screening in the community, saliva samples provide undeniable advantages in comparison with nasopharyngeal swabs: collection is not invasive, it is inexpensive and requires only a sterile container, it can be self-performed easily, and it reduces the risk of exposure to infectious aerosols for health workers [20, 21]. Among the 23 studies included in the meta-analysis from Tsang et al [4], 13 evaluated the diagnostic performance of saliva samples. The overall PPA was 85% (95% CI, 75%–93%), although heterogeneity was observed, with 3 studies showing lower values (31%, 73%, and 79%) linked to low COVID-19 prevalence and/or inclusion of asymptomatic participants. The NPA was >90% for all the studies. In Laos, routine SARS-CoV-2 diagnosis and surveillance were based on nasopharyngeal swabs collected in VTM and sent to central laboratories in Vientiane for detection by RT-qPCR until rapid diagnostic tests became available. In our study, we found a high PPA of saliva samples with swab samples in VTM: 89.3% (95% CI, 85.2%–92.6%). The NPA was lower than that calculated in the studies of the meta-analysis from Tsang et al. Yet, in those studies, SARS-CoV-2 prevalence was lower (range, 4.3%–38.6%) than in our study (60.3%). The NPA calculated for samples collected in a period or site with low SARS-CoV-2 was >99.0% (2023 and LNT, respectively). Low negative agreement could also be related to the variable quality of swab collection, as suggested by Wyllie et al, who reported that RNaseP polymerase chain reaction results were more variable in nasopharyngeal samples than saliva [22]. Regardless, our findings show good agreement between saliva and swab samples in VTM for the detection of SARS-CoV-2 by RT-qPCR.

Additional challenges that low-resource settings such as Laos face for the implementation of nationwide surveillance or cluster investigation in remote areas is a distinct lack of access to reference laboratory facilities, which are often only available in larger cities. Dry ice shipments are typically not available and can be very expensive. The use of filter paper for blood or whole blood collection and storage provides good performance for subsequent viral RNA detection and characterization of other pathogens [8–10]. In our study, after drying and storage for 1 week at room temperature, there was good overall agreement in comparison with swabs collected in VTM for SARS-CoV-2 detection. In a study of 56 participants, Khan and Roopa found a bit higher concordance between the samples (κ = 0.82 [95% CI, 0.67–0.96]; overall percent agreement, 91.1% [95% CI, 80.4%–97.0%]); however, the Cq value threshold for positivity was 35 and all positive samples except 1 had a Cq ≤ 33. In addition, there was no mention of storing the dry swabs at room temperature [16]. As expected, a decrease in sensitivity was observed with higher Cq values from dry swabs in comparison with swabs in VTM. Yet, this had limited impact as shown by a PPA >80%, which is higher than the performance of an antigen rapid diagnostic test, with an overall PPA of 76.3% estimated in a meta-analysis including 194 clinical accuracy studies [23]. In addition, as swab in VTM is an imperfect standard, with some positive participants not being detected, the performance of dry swabs could be underestimated.

In our study, SARS-CoV-2 RNA was successfully purified from dry saliva on filter paper and then detected by RT-qPCR. There was a decrease in sensitivity when compared with neat saliva, with higher Cq values (Supplementary Figure 1*C*) and moderate PPA (73.6%; 95% CI, 68.1%–78.6%). This is similar to that in studies comparing dengue virus detection by RT-qPCR from dried serum on filter paper and from neat serum, with a PPA of 78% (95% CI, 55%–91%) reported by Curren et al during an outbreak in American Samoa [24] and 70.8% (95% CI, 55.9%–83.0%) with samples from hospitalized patients in Laos [8]. In contrast, whereas the NPAs were close to 100%, our study yielded an NPA of 84.5%. This may be due to the viscosity of some saliva samples, which can affect the efficiency of the RNA purification process, but also to the quality of saliva samples that were stored at 4 °C and processed with some delay after collection, up to 7 days at 2 to 8 °C before processing. Our results showed lower performance of the use of dry saliva on filter paper as compared with dry swabs (PPA, 73.8% [95% CI, 68.3%–78.8%] vs 84.8% [95% CI, 80.2%–88.8%]; *P* = .001).

Our study has some limitations. Saliva samples were stored at 4 °C at provincial sites before transportation to the laboratory in Vientiane, though in some cases this might mirror real-world conditions during a pandemic. Swabs collected in VTM were processed after some delay. However, samples were frozen immediately after collection, and a cool box was used with a temperature logger for transportation; therefore, we believe that sample degradation before processing was minimal. Different extraction kits were used for the processing of swabs in VTM, although the performance of the 2 kits is similar, which limits a potential impact on the results. The study was conducted at 3 sites; as such, we cannot exclude some variability in sampling methods among them. This study did not include asymptomatic presentation, and we did not assess the effect of disease severity. Finally, children were not included in the study.

Our findings suggest that dry swabs could be a viable alternative for sample collection and permit easy shipping at room temperature for subsequent viral SARS-CoV-2 RNA purification and molecular investigation in patients presenting with acute respiratory infection. This could be of particular interest in low-resource settings for cluster investigations in remote areas or for sentinel community biosurveillance. A similar approach is used in community-based studies with self-collection of nasal dry swab to evaluate vaccine effectiveness [25]. There may be other situations where saliva could be a preferred sample—for example, to reduce the risk of sampling highly infectious agents by health care workers or in patients who refuse nasopharyngeal swab. Additional investigations would be necessary to assess the impact of longer storage at higher temperatures of the dry swab on the detection of SARS-CoV-2. While dried saliva on filter paper did not show as good performance as either VTM or saliva samples, some improvement could be provided by the use of smaller-sized standardized filter paper and the evaluation of the impact of saliva sample viscosity.

Collection of dry nasopharyngeal swab and saliva samples is a useful tool to consider for the rapid implementation of large-scale surveillance of SARS-CoV-2 in symptomatic patients in remote areas, which could be extrapolated to other respiratory targets and help make public health monitoring of respiratory viruses more efficient and cost-effective.

## Supplementary Material

ofae433_Supplementary_Data

## Supplementary Data

Supplementary materials are available at *Open Forum Infectious Diseases* online. Consisting of data provided by the authors to benefit the reader, the posted materials are not copyedited and are the sole responsibility of the authors, so questions or comments should be addressed to the corresponding author.

## Notes

***Acknowledgments.*** We thank all the participants of this study; the directors and staff of the Xiengkhuang Provincial Hospital, Attapeu Provincial Hospital, and Luang Namtha Provincial Hospital; the staff of the Microbiology Laboratory in Mahosot Hospital for their technical help and supports; the Lao-Oxford-Mahosot Hospital-Wellcome Trust Research Unit staff supporting participant recruitment at hospital sites; Touxiong Yiaye and Phatsalin Keomoukda at Xiengkhuang; Soulichanya Phoutthavong and Sompong Thammavong at Attapeu; Touy Phommavong and Thongsavanh Lathsachack at Luang Namtha; and Matthew Robinson and Elizabeth Batty for supporting whole genome sequencing.

***Author contributions.*** Design and conception of the project: A. D.-P., E. A. A. Data collection: D. C., L. B., V. P., S. B., H. S. Analysis: L. B., B. S. Interpretation of the analysis: B. S., A. D.-P., S. P. Management: K. P., D. C., L. B., M. M., M. V., E. A. A. Resources: N. C. V. C., A. G. L., M. M., M. V., E. A. A., A. D.-P. Writing of the manuscript: A. D.-P., E. A. A., B. S., N. C. V. C., A. G. L.

***Disclaimer.*** The views expressed in this article reflect the results of research conducted by the authors and do not necessarily reflect the official policy or position of the Department of the Navy, Department of Defense, or US government. A. G. L. (CAPT, MC, USN) and N. C. V. C. (LCDR, MSC, USN) are military service members of the US government. This work was prepared as part of their official duties. Title 17 USC 105 provides that “copyright protection under this title is not available for any work of the United States Government.” Title 17 USC 101 defines a US government work as work prepared by a military service member or employee of the US government as part of that person's official duties.

***Open access.*** For the purpose of open access, the authors have applied a CC BY public copyright licence to any author accepted manuscript version arising from this submission.

***Financial support*.** The respiratory virus surveillance study was funded by the Defense Health Agency, United States as part of the Department of Defense Global Emerging Infections and Surveillance (project P0153_21_N2, work unit D0016), the Institute of Research for Development, and Aix-Marseille University. This study was funded by the MORU award program from Wellcome Trust: Institutional Translational Partnership Awards–Thailand Major Overseas Programme (grant COV2020009/007/707). The Lao-Oxford-Mahosot Hospital-Wellcome Trust Research Unit is part of the MORU Tropical Health Network, funded by the Wellcome Trust. This research was funded in part by the Wellcome Trust (grant 220211/Z/20/Z).
