# Supplementary material for: Dry Swabs and Dried Saliva as Alternative Samples for SARS-CoV-2 Detection in Remote Areas in Lao PDR
Source: Open Forum Infect Dis. 2024 Jul 23;11(8):ofae433. doi: 10.1093/ofid/ofae433 (PMC11322834; doi:10.1093/ofid/ofae433)
Supplement: ofae433_Supplementary_Data [file ofae433_supplementary_data.docx]

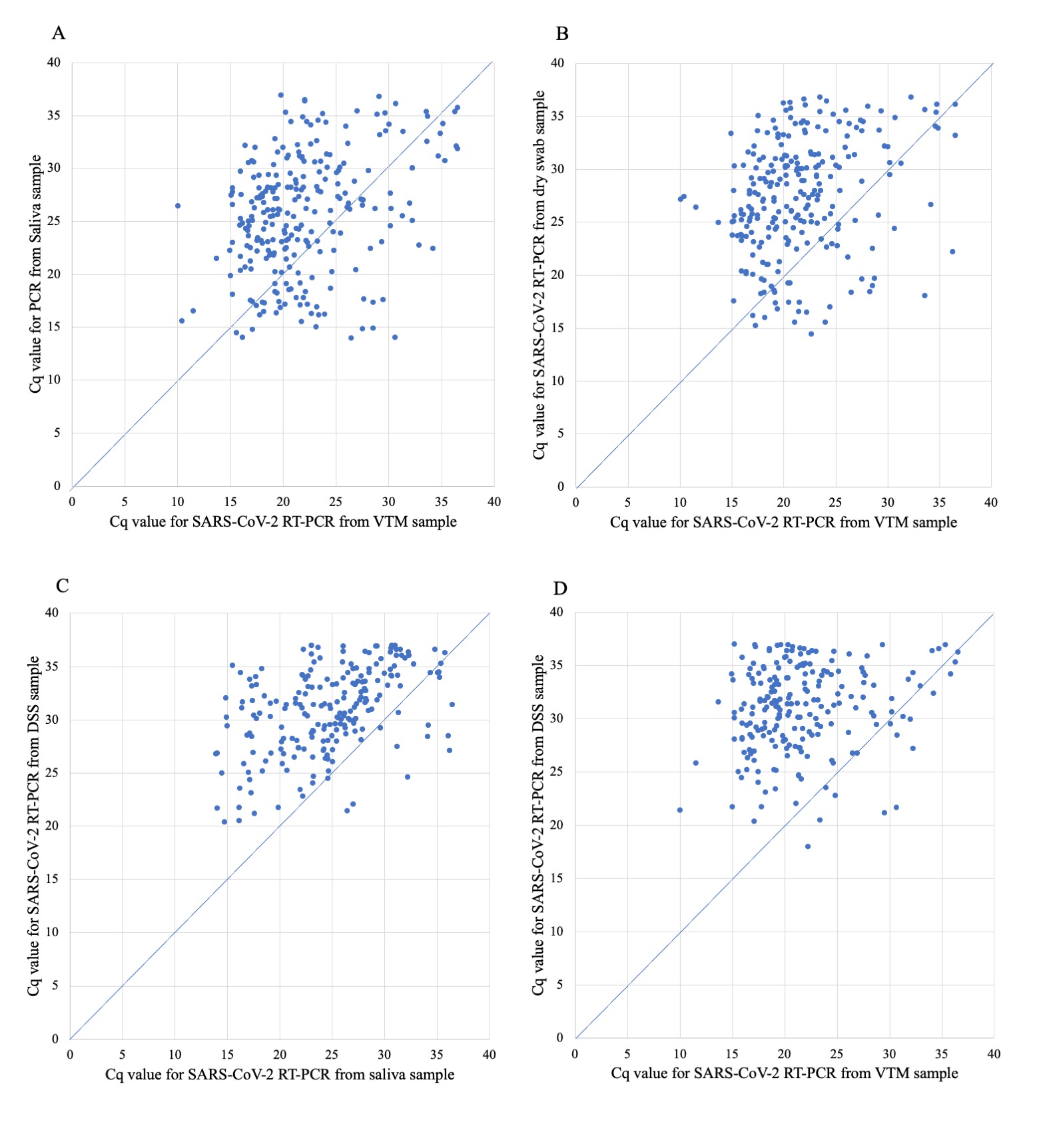


**Figure S1**: Scatter plot comparing SARS-CoV-2 RT-qPCR Cq values for the different samples. A: Saliva and VTM samples. B: Dry swab and VTM samples. C: DSS and saliva samples. D: DSS and VTM samples. VTM: Nasopharyngeal/oropharyngeal swabs in virus transport medium. DSS: dried saliva spotted on filter paper.


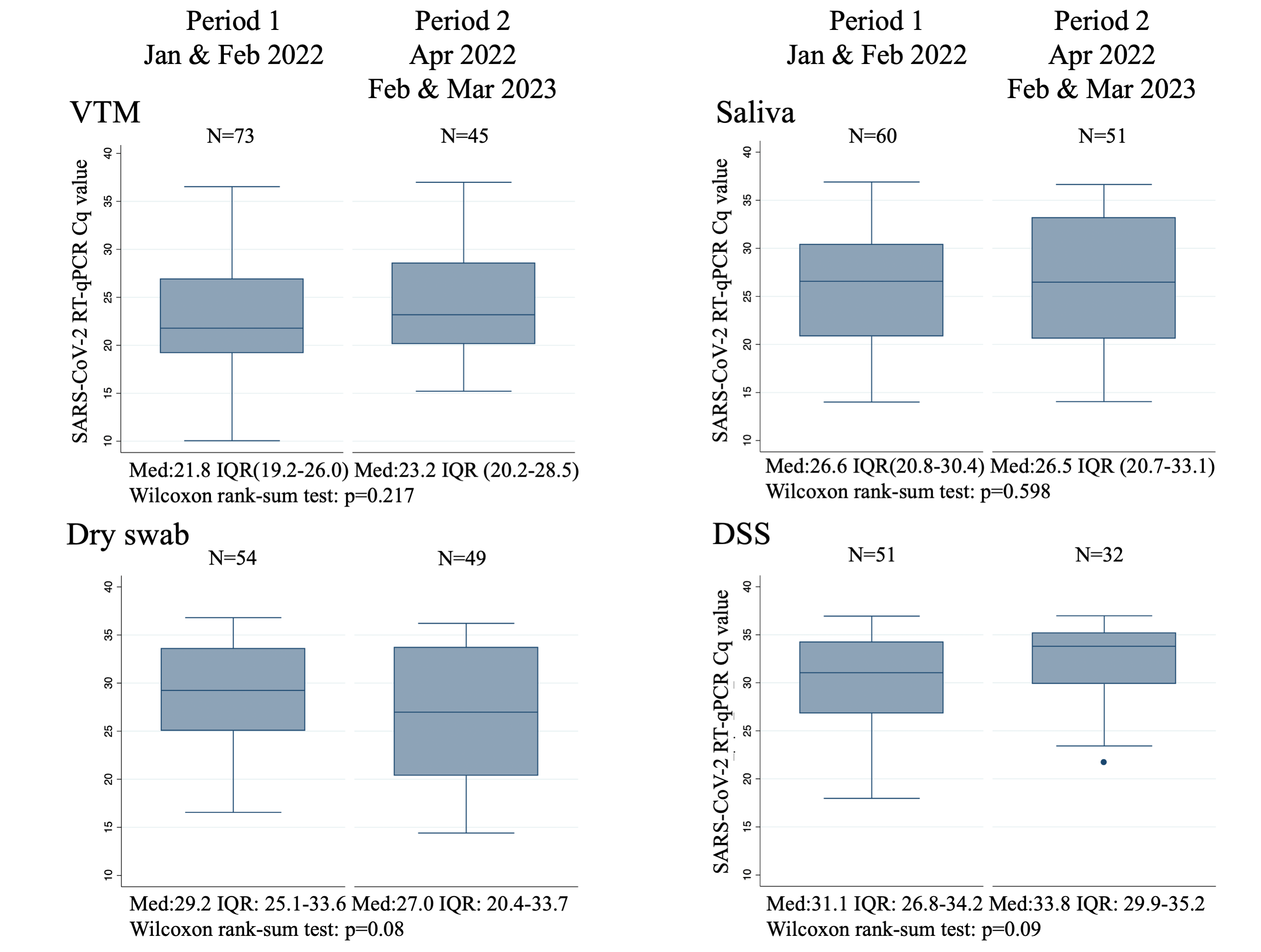


**Figure S2**: Boxplots of SARS-CoV-2 RT-qPCR Cq values during period 1 and period 2 for VTM, saliva, dry swab and DSS samples.

**Table S1** : Socio-demographic and clinical data of participants.

|  | Total  N=479 | Attapeu  N=148 | Luang-Namtha  N=96 | Xiengkhuang N=235 |
| --- | --- | --- | --- | --- |
| Gender |  |  |  |  |
| Male, n (%)  Female, n (%) | 232 (48.4)  247 (51.6) | 75 (50.7)  73 (49.3) | 43 (44.8)  53 (55.2) | 114 (48.5)  121 (51.5) |
| Age, median (IQR) | 35 (27-46) | 35 (27-42) | 38 (29-54) | 34 (26-45) |
| Number of days of illness, median (IQR) | 2 (3-4) | 2 (1-4) | 2 (1-3) | 3 (2-4) |
| Sore throat, n (%) | 408 (85.2) | 96 (64.9) | 81 (84.4) | 231 (98.3) |
| Runny nose, n (%) | 344 (71.8) | 93 (62.8) | 56 (58.3) | 195 (82.9) |
| Cough, n(%) | 443 (92.5) | 114 (77.0) | 95 (99.0) | 234 (99.6) |
| Shortness of breath, n (%) | 41 (8.6) | 2 (1.4) | 18 (18.8) | 21 (8.9) |
| Headache, n (%) | 350 (73.1) | 92 (62.2) | 31 (32.3) | 227 (96.6) |
| Loss of smell, n (%) | 152 (31.7) | 33 (22.3) | 0 | 119 (50.6) |
| Loss of taste, n (%) | 50 (10.4) | 18 (12.2) | 0 | 32 (13.6) |
| History of COVID-19 vaccination*, n (%) | 400 (83.5) | 135 (91.2) | 67 (69.8) | 198 (84.3) |
| Type of the first COVID-19 vaccine received |  |  |  |  |
| Sinopharm | 253 (63.3) | 77 (57.0) | 46 (68.7) | 130 (65.7) |
| Janssens (Johnson & Johnson) | 80 (20.0) | 30 (22.2) | 14 (20.9) | 36 (18.2) |
| AstraZeneca/University of Oxford (Covishield in India) | 43 (10.8) | 20 (14.8) | 7 (10.5) | 16 (8.1) |
| Sinovac | 4 (1.0) | 4 (3.0) | 0 | 0 |
| Pfizer/BioNTech | 1 (0.3) | 0 | 0 | 1 (0.5) |
| Sputnik V | 1 (0.3) | 0 | 0 | 1 (0.5) |
| Unknown | 18 (4.5) | 4 (3.0) | 0 | 14 (7.1) |
| SARS-CoV-2 VTM positive, n (%) | 288 (60.1) | 99 (66.9) | 33 (34.4) | 156 (66.4) |

VTM: Nasopharyngeal/oropharyngeal swabs in virus transport medium.

*Participant has received at least one COVID-19 vaccination.

**Table S2** : Results of comparison of SARS-CoV-2 RT-qCR results for the different samples at each study site.

|  | | **Attapeu**  **N=148** | | | | **Luang-Namtha**  **N=96** | | | **Xiengkhuang**  **N=235** | | | | |
| --- | --- | --- | --- | --- | --- | --- | --- | --- | --- | --- | --- | --- | --- |
|  | | **VTM** | | | **Total** | **VTM** | | **Total** | **VTM** | | | **Total** | |
|  |  | **Pos** | **Neg** | |  | **Pos** | **Neg** |  | **Pos** | **Neg** | |  |  |
| **Saliva** | **Positive** | 95 | 12 | | 107 | 29 | 5 | 34 | 133 | 12 | | 145 | |
|  | **Negative** | 4 | 37 | | 41 | 4 | 58 | 62 | 23 | 67 | | 90 | |
| **Total** | | 99 | 49 | | 148 | 33 | 63 | 96 | 156 | 79 | | 235 | |
| OPA :(95%CI):  PPA (95%CI):  NPA (95%CI): Kappa (95%CI): | | 89.2% (83.0-93.7)  96.0% (90.0-98.9)  75.5% (61.1-86.7)  0.75 (0.63-0.86) | | | | 90.6% (82.9-95.6)  87.9% (71.8-96.6)  92.1% (82.4-97.4)  0.79 (0.67-0.92) | | | 85.1% (79.9-89.4)  85.3% (78.7-90.4)  84.8% (75.0-91.9)  0.68 (0.58-0.78) | | | | |
| **Dry swab** | **Positive** | 90 | | 7 | 97 | 28 | 6 | 34 | 127 | | 12 | | 139 |
|  | **Negative** | 9 | | 42 | 51 | 5 | 57 | 62 | 29 | | 67 | | 96 |
| **Total** | | 99 | | 49 | 148 | 33 | 63 | 96 | 156 | | 79 | | 235 |
| OPA :(95%CI):  PPA (95%CI):  NPA (95%CI): Kappa (95%CI): | | 89.2% (83.0-93.7)  90.9% (83.4-95.8)  85.7% (72.8-94.1)  0.76 (0.65-0.87) | | | | 88.5% (80.4-94.1)  84.8% (68.1-94.9)  90.5% (80.4-96.4)  0.75 (0.61-0.89) | | | 82.6% (77.1-87.2)  81.4% (74.4-87.2)  84.8% (75.0-91.9)  0.63 (0.53-0.73) | | | | |
| **DSS** | **Positive** | 83 | | 9 | 92 | 25 | 8 | 33 | 106 | | 11 | | 117 |
|  | **Negative** | 16 | | 40 | 56 | 8 | 55 | 63 | 50 | | 68 | | 118 |
| **Total** | | 99 | | 49 | 148 | 33 | 63 | 96 | 156 | | 79 | | 235 |
| OPA :(95%CI):  PPA (95%CI):  NPA (95%CI): Kappa (95%CI): | | 83.1% (76.1-88.8)  83.8% (75.1-90.5)  81.6% (68.0-91.2)  0.63 (0.50-0.76) | | | | 83.3% (74.4-90.2)  75.8% (57.7-88.9)  65.6% (55.2-75.0)  0.63 (0.47-0.80) | | | 74.0% (67.9-79.5)  67.9% (60.0-75.2)  86.1% (76.5-92.8)  0.48 (0.38-0.59) | | | | |
|  | | **Saliva** | | | **Total** | **Saliva** | | **Total** | **Saliva** | | | | **Total** |
|  | | **Pos** | | **Neg** |  | **Pos** | **Neg** |  | **Pos** | | **Neg** | |  |
| **DSS** | **Positive** | 85 | | 7 | 92 | 23 | 10 | 33 | 104 | | 13 | | 117 |
|  | **Negative** | 22 | | 34 | 56 | 11 | 52 | 63 | 41 | | 77 | | 118 |
| **Total** | | 107 | | 41 | 148 | 34 | 62 | 96 | 145 | | 90 | | 235 |
| OPA :(95%CI):  PPA (95%CI):  NPA (95%CI): Kappa (95%CI): | | 80.4% (73.1-86.5)  79.4% (70.5-86.6)  82.9% (67.9-92.8)  0.56 (0.42-0.70) | | | | 78.1% (68.5- 85.9)  67.6% (49.5-82.6)  83.9% (72.3-92.0)  0.52 (0.34-0.70) | | | 77.0% (71.1-82.2)  71.7% (63.7-78.9)  85.6% (76.6-92.1)  0.54 (0.44-0.65) | | | | |

OPA : overall percent agreement ; PPA: positive percent agreement; NPA: negative percent agreement; DSS: dried saliva spotted on filter paper. VTM: Nasopharyngeal/oropharyngeal swabs in virus transport medium.

**Table S3** : Results of comparison of SARS-CoV-2 RT-qCR results for the different samples for patients with less than 5 days of illness at sample collection and patients with 5 to less than 10 days of illness at sample collection.

|  | | **Less than 5 days of illness**  **N=380** | | | | **5 to 9 days of illness**  **N=83** | | |
| --- | --- | --- | --- | --- | --- | --- | --- | --- |
|  | | **VTM** | | | **Total** | **VTM** | | **Total** |
|  |  | **Pos** | **Neg** | |  | **Pos** | **Neg** |  |
| **Saliva** | **Positive** | 220 | 19 | | 239 | 30 | 8 | 38 |
|  | **Negative** | 23 | 118 | | 141 | 6 | 39 | 45 |
| **Total** | | 243 | 137 | | 380 | 36 | 47 | 83 |
| OPA :(95%CI):  PPA (95%CI):  NPA (95%CI): Kappa (95%CI): | | 88.9% (85.4-91.9)  90.5% (86.1-93.9)  86.1% (79.2-91.4)  0.76 (0.69-0.83) | | | | 83.1% (73.3-90.5)  83.3% (67.2-93.6)  83.0% (69.2-92.4)  0.66 (0.50-0.82) | | |
| **Dry swab** | **Positive** | 213 | | 16 | 229 | 26 | 8 | 34 |
|  | **Negative** | 30 | | 121 | 151 | 10 | 39 | 49 |
| **Total** | | 243 | | 137 | 380 | 36 | 47 | 83 |
| OPA :(95%CI):  PPA (95%CI):  NPA (95%CI): Kappa (95%CI): | | 87.9% (84.2-91.0)  87.7% (82.8-91.5)  88.3% (81.7-93.2)  0.74 (0.67-0.81) | | | | 78.3% (67.9-86.6)  72.2% (54.8-85.8)  83.0% (69.2-92.4)  0.56 (0.38-0.74) | | |
| **DSS** | **Positive** | 184 | | 20 | 204 | 24 | 7 | 31 |
|  | **Negative** | 59 | | 117 | 176 | 12 | 40 | 52 |
| **Total** | | 243 | | 137 | 380 | 36 | 47 | 83 |
| OPA :(95%CI):  PPA (95%CI):  NPA (95%CI): Kappa (95%CI): | | 79.2% (74.8-83.2)  75.7% (69.8-81.0)  85.4% (78.4-90.8)  0.58 (0.49-0.66) | | | | 77.1% (66.6-85.6)  66.7% (49.0-81.4)  85.1% (71.7-93.8)  0.53 (0.34-0.71) | | |
|  | | **Saliva** | | | **Total** | **Saliva** | | **Total** |
|  | | **Pos** | | **Neg** |  | **Pos** | **Neg** |  |
| **DSS** | **Positive** | 187 | | 17 | 204 | 21 | 10 | 31 |
|  | **Negative** | 52 | | 124 | 176 | 17 | 35 | 52 |
| **Total** | | 239 | | 141 | 380 | 38 | 45 | 83 |
| OPA :(95%CI):  PPA (95%CI):  NPA (95%CI): Kappa (95%CI): | | 81.8% (77.6-85.6)  78.2% (72.5-83.3)  87.9% (81.4-92.8)  0.63 (0.55-0.71) | | | | 67.5% (56.3-77.4)  55.3% (38.3-71.4)  77.8% (62.9-88.8)  0.34 (0.13-0.54) | | |

OPA : overall percent agreement ; PPA: positive percent agreement; NPA: negative percent agreement; DSS: dried saliva spotted on filter paper. VTM: Nasopharyngeal/oropharyngeal swabs in virus transport medium.

**Table S4**: Genbank and GISAID accession numbers for the SARS-COV-2 whole genome sequences included in the study.

| Genbank | GISAID | Sample date | Variant | Sample name |
| --- | --- | --- | --- | --- |
| ON922849 | EPI_ISL_13351937 | 21-Feb-22 | Delta | ATP00925-0031 |
| ON922848 | EPI_ISL_13351936 | 25-Feb-22 | Delta | ATP00925-0046 |
| ON922844 | EPI_ISL_13351932 | 28-Feb-22 | Delta | ATP00925-0051 |
| ON922845 | EPI_ISL_13351933 | 01-Mar-22 | Delta | ATP00925-0054 |
| ON922850 | EPI_ISL_13351938 | 02-Mar-22 | Delta | ATP00925-0056 |
| ON922851 | EPI_ISL_13351939 | 02-Mar-22 | Delta | ATP00925-0057 |
| ON922852 | EPI_ISL_13351940 | 04-Mar-22 | Delta | ATP00925-0065 |
| OP159582 | EPI_ISL_13094355 | 09-Mar-22 | Delta | ATP00925-0070 |
| OP159583 | EPI_ISL_13094356 | 09-Mar-22 | Omicron | ATP00925-0071 |
| OP159584 | EPI_ISL_13094357 | 09-Mar-22 | Delta | ATP00925-0072 |
| OP159585 | EPI_ISL_13094358 | 10-Mar-22 | Omicron | ATP00925-0074 |
| OP159586 | EPI_ISL_13094359 | 10-Mar-22 | Delta | ATP00925-0075 |
| OP159587 | EPI_ISL_13094360 | 10-Mar-22 | Omicron | ATP00925-0076 |
| OP159588 | EPI_ISL_13094361 | 11-Mar-22 | Delta | ATP00925-0078 |
| OP159589 | EPI_ISL_13094362 | 11-Mar-22 | Delta | ATP00925-0079 |
| OP159420 | EPI_ISL_12983528 | 14-Mar-22 | Delta | ATP00925-0082 |
| OP159421 | EPI_ISL_12983529 | 14-Mar-22 | Omicron | ATP00925-0083 |
| OP159422 | EPI_ISL_12983530 | 15-Mar-22 | Omicron | ATP00925-0085 |
| OP159423 | EPI_ISL_12983531 | 15-Mar-22 | Delta | ATP00925-0087 |
| OP159424 | EPI_ISL_12983532 | 16-Mar-22 | Omicron | ATP00925-0089 |
| OP159425 | EPI_ISL_12983533 | 16-Mar-22 | Omicron | ATP00925-0091 |
| OP159426 | EPI_ISL_12983534 | 17-Mar-22 | Delta | ATP00925-0092 |
| OP159427 | EPI_ISL_12983535 | 17-Mar-22 | Delta | ATP00925-0093 |
| OP159428 | EPI_ISL_12983536 | 17-Mar-22 | Delta | ATP00925-0095 |
| OP159429 | EPI_ISL_12983537 | 17-Mar-22 | Delta | ATP00925-0096 |
| OP159430 | EPI_ISL_12983538 | 18-Mar-22 | Omicron | ATP00925-0097 |
| OP159431 | EPI_ISL_12983539 | 18-Mar-22 | Omicron | ATP00925-0098 |
| OP159432 | EPI_ISL_12983540 | 18-Mar-22 | Omicron | ATP00925-0100 |
| OP132721 | EPI_ISL_12987033 | 21-Mar-22 | Omicron | ATP00925-0102 |
| OP132722 | EPI_ISL_12987034 | 21-Mar-22 | Omicron | ATP00925-0103 |
| OP132723 | EPI_ISL_12987035 | 21-Mar-22 | Delta | ATP00925-0105 |
| OP132724 | EPI_ISL_12987036 | 22-Mar-22 | Omicron | ATP00925-0106 |
| OP132725 | EPI_ISL_12987037 | 22-Mar-22 | Omicron | ATP00925-0107 |
| OP132726 | EPI_ISL_12987038 | 22-Mar-22 | Omicron | ATP00925-0108 |
| OP132727 | EPI_ISL_12987039 | 22-Mar-22 | Omicron | ATP00925-0109 |
| OP132728 | EPI_ISL_12987040 | 22-Mar-22 | Omicron | ATP00925-0110 |
| OP132729 | EPI_ISL_12987041 | 23-Mar-22 | Delta | ATP00925-0111 |
| OP132730 | EPI_ISL_12987042 | 23-Mar-22 | Delta | ATP00925-0112 |
| OQ028413 | EPI_ISL_15973657 | 23-Mar-22 | Omicron | ATP00925-0113 |
| OP132771 | EPI_ISL_13092022 | 23-Mar-22 | Delta | ATP00925-0114 |
| OP132731 | EPI_ISL_12987043 | 24-Mar-22 | Omicron | ATP00925-0118 |
| OP132732 | EPI_ISL_12987044 | 24-Mar-22 | Omicron | ATP00925-0119 |
| OP132734 | EPI_ISL_12987046 | 25-Mar-22 | Omicron | ATP00925-0121 |
| OP132735 | EPI_ISL_12987047 | 25-Mar-22 | Omicron | ATP00925-0122 |
| OP132736 | EPI_ISL_12987048 | 25-Mar-22 | Omicron | ATP00925-0123 |
| OP159461 | EPI_ISL_13091936 | 28-Mar-22 | Omicron | ATP00925-0126 |
| OP159462 | EPI_ISL_13091937 | 28-Mar-22 | Omicron | ATP00925-0127 |
| OQ028390 | EPI_ISL_15973634 | 28-Mar-22 | Omicron | ATP00925-0128 |
| OP159463 | EPI_ISL_13091938 | 29-Mar-22 | Omicron | ATP00925-0129 |
| OP159464 | EPI_ISL_13091939 | 29-Mar-22 | Omicron | ATP00925-0131 |
| OQ028391 | EPI_ISL_15973635 | 29-Mar-22 | Omicron | ATP00925-0132 |
| OP159465 | EPI_ISL_13091940 | 30-Mar-22 | Omicron | ATP00925-0134 |
| OP159467 | EPI_ISL_13091942 | 30-Mar-22 | Omicron | ATP00925-0138 |
| OP159468 | EPI_ISL_13091943 | 31-Mar-22 | Omicron | ATP00925-0139 |
| OQ028392 | EPI_ISL_15973636 | 31-Mar-22 | Omicron | ATP00925-0140 |
| OQ028393 | EPI_ISL_15973637 | 31-Mar-22 | Omicron | ATP00925-0141 |
| OP159469 | EPI_ISL_13091944 | 31-Mar-22 | Omicron | ATP00925-0142 |
| OP159470 | EPI_ISL_13091945 | 31-Mar-22 | Omicron | ATP00925-0143 |
| OP159471 | EPI_ISL_13091946 | 01-Apr-22 | Omicron | ATP00925-0144 |
| OP159472 | EPI_ISL_13091947 | 01-Apr-22 | Omicron | ATP00925-0145 |
| OQ028394 | EPI_ISL_15973638 | 01-Apr-22 | Omicron | ATP00925-0146 |
| OP159473 | EPI_ISL_13091948 | 01-Apr-22 | Omicron | ATP00925-0147 |
| OP159474 | EPI_ISL_13091949 | 01-Apr-22 | Omicron | ATP00925-0148 |
| OP159497 | EPI_ISL_13091972 | 04-Apr-22 | Omicron | ATP00925-0149 |
| OQ028400 | EPI_ISL_15973644 | 04-Apr-22 | Omicron | ATP00925-0150 |
| OP159498 | EPI_ISL_13091973 | 04-Apr-22 | Omicron | ATP00925-0152 |
| OP159499 | EPI_ISL_13091974 | 04-Apr-22 | Omicron | ATP00925-0153 |
| OP159500 | EPI_ISL_13091975 | 05-Apr-22 | Omicron | ATP00925-0155 |
| OP159501 | EPI_ISL_13091976 | 05-Apr-22 | Omicron | ATP00925-0156 |
| OP159502 | EPI_ISL_13091977 | 05-Apr-22 | Omicron | ATP00925-0158 |
| OQ028401 | EPI_ISL_15973645 | 06-Apr-22 | Omicron | ATP00925-0159 |
| OP159503 | EPI_ISL_13091978 | 06-Apr-22 | Omicron | ATP00925-0160 |
| OP159504 | EPI_ISL_13091979 | 07-Apr-22 | Omicron | ATP00925-0162 |
| OP159506 | EPI_ISL_13091981 | 07-Apr-22 | Omicron | ATP00925-0164 |
| OQ028402 | EPI_ISL_15973646 | 08-Apr-22 | Omicron | ATP00925-0166 |
| OP159507 | EPI_ISL_13091982 | 08-Apr-22 | Omicron | ATP00925-0167 |
| OP159435 | EPI_ISL_12983543 | 15-Mar-22 | Omicron | LNT00820-0438 |
| OP159436 | EPI_ISL_12983544 | 15-Mar-22 | Omicron | LNT00820-0439 |
| OP159437 | EPI_ISL_12983545 | 15-Mar-22 | Omicron | LNT00820-0441 |
| OP159438 | EPI_ISL_12983546 | 16-Mar-22 | Omicron | LNT00820-0444 |
| OP159439 | EPI_ISL_12983547 | 16-Mar-22 | Omicron | LNT00820-0445 |
| OP159407 | EPI_ISL_12983515 | 16-Mar-22 | Omicron | LNT00820-0447 |
| OP159440 | EPI_ISL_12983548 | 17-Mar-22 | Omicron | LNT00820-0450 |
| OP159441 | EPI_ISL_12983549 | 17-Mar-22 | Omicron | LNT00820-0451 |
| OP159442 | EPI_ISL_12983550 | 17-Mar-22 | Omicron | LNT00820-0452 |
| OP159408 | EPI_ISL_12983516 | 18-Mar-22 | Omicron | LNT00820-0454 |
| OP159443 | EPI_ISL_12983551 | 18-Mar-22 | Omicron | LNT00820-0455 |
| OP159444 | EPI_ISL_12983552 | 18-Mar-22 | Omicron | LNT00820-0456 |
| OP159445 | EPI_ISL_12983553 | 18-Mar-22 | Omicron | LNT00820-0457 |
| OP159475 | EPI_ISL_13091950 | 21-Mar-22 | Omicron | LNT00820-0458 |
| OP159476 | EPI_ISL_13091951 | 21-Mar-22 | Omicron | LNT00820-0459 |
| OP159478 | EPI_ISL_13091953 | 21-Mar-22 | Omicron | LNT00820-0461 |
| OP159479 | EPI_ISL_13091954 | 21-Mar-22 | Omicron | LNT00820-0462 |
| OP159482 | EPI_ISL_13091957 | 22-Mar-22 | Omicron | LNT00820-0465 |
| OP159483 | EPI_ISL_13091958 | 22-Mar-22 | Omicron | LNT00820-0466 |
| OP132769 | EPI_ISL_13092020 | 06-Apr-22 | Omicron | LNT00820-0488 |
| OP132770 | EPI_ISL_13092021 | 11-Apr-22 | Omicron | LNT00820-0498 |
| OQ028282 | EPI_ISL_15656014 | 01-Feb-22 | Delta | XK00819-0723 |
| OP474028 | EPI_ISL_15022088 | 07-Feb-22 | Delta | XK00819-0737 |
| OQ028286 | EPI_ISL_15656010 | 07-Feb-22 | Delta | XK00819-0738 |
| OQ028287 | EPI_ISL_15656009 | 07-Feb-22 | Delta | XK00819-0739 |
| OQ028388 | EPI_ISL_15973632 | 08-Feb-22 | Delta | XK00819-0740 |
| OP474029 | EPI_ISL_15022089 | 08-Feb-22 | Delta | XK00819-0741 |
| OP474030 | EPI_ISL_15022090 | 08-Feb-22 | Delta | XK00819-0743 |
| OQ028288 | EPI_ISL_15656008 | 08-Feb-22 | Delta | XK00819-0744 |
| OQ028337 | EPI_ISL_15655959 | 09-Feb-22 | Delta | XK00819-0746 |
| OQ028338 | EPI_ISL_15655958 | 09-Feb-22 | Delta | XK00819-0748 |
| OQ028339 | EPI_ISL_15655957 | 09-Feb-22 | Delta | XK00819-0749 |
| OQ028340 | EPI_ISL_15655956 | 10-Feb-22 | Delta | XK00819-0750 |
| OQ028341 | EPI_ISL_15655955 | 10-Feb-22 | Delta | XK00819-0751 |
| OQ028342 | EPI_ISL_15655954 | 11-Feb-22 | Delta | XK00819-0755 |
| OQ028343 | EPI_ISL_15655953 | 11-Feb-22 | Delta | XK00819-0756 |
| OQ028344 | EPI_ISL_15655952 | 11-Feb-22 | Delta | XK00819-0757 |
| OQ028345 | EPI_ISL_15655951 | 11-Feb-22 | Delta | XK00819-0758 |
| OQ028346 | EPI_ISL_15655950 | 14-Feb-22 | Delta | XK00819-0760 |
| OQ028389 | EPI_ISL_15973633 | 14-Feb-22 | Delta | XK00819-0762 |
| OQ028347 | EPI_ISL_15655949 | 15-Feb-22 | Delta | XK00819-0763 |
| OQ028426 | EPI_ISL_15973670 | 15-Feb-22 | Delta | XK00819-0765 |
| OQ028461 | EPI_ISL_16003856 | 15-Feb-22 | Delta | XK00819-0766 |
| OQ028348 | EPI_ISL_15655948 | 16-Feb-22 | Delta | XK00819-0769 |
| OQ028349 | EPI_ISL_15655947 | 16-Feb-22 | Delta | XK00819-0770 |
| OQ028350 | EPI_ISL_15655946 | 18-Feb-22 | Delta | XK00819-0780 |
| OQ028351 | EPI_ISL_15655945 | 23-Feb-22 | Delta | XK00819-0783 |
| OQ028427 | EPI_ISL_15973671 | 23-Feb-22 | Delta | XK00819-0784 |
| OQ028428 | EPI_ISL_15973672 | 23-Feb-22 | Delta | XK00819-0785 |
| ON922839 | EPI_ISL_13351927 | 24-Feb-22 | Delta | XK00819-0790 |
| ON922840 | EPI_ISL_13351928 | 24-Feb-22 | Delta | XK00819-0792 |
| ON922841 | EPI_ISL_13351929 | 25-Feb-22 | Delta | XK00819-0793 |
| ON922842 | EPI_ISL_13351930 | 25-Feb-22 | Delta | XK00819-0795 |
| OP132700 | EPI_ISL_12982424 | 01-Mar-22 | Omicron | XK00819-0802 |
| OP132699 | EPI_ISL_12982423 | 01-Mar-22 | Delta | XK00819-0803 |
| OP132708 | EPI_ISL_12982432 | 01-Mar-22 | Delta | XK00819-0806 |
| OP132703 | EPI_ISL_12982427 | 02-Mar-22 | Delta | XK00819-0807 |
| OP132707 | EPI_ISL_12982431 | 02-Mar-22 | Delta | XK00819-0810 |
| OP132704 | EPI_ISL_12982428 | 03-Mar-22 | Delta | XK00819-0812 |
| OP132701 | EPI_ISL_12982425 | 03-Mar-22 | Omicron | XK00819-0815 |
| OP132706 | EPI_ISL_12982430 | 04-Mar-22 | Delta | XK00819-0817 |
| OP132705 | EPI_ISL_12982429 | 04-Mar-22 | Delta | XK00819-0818 |
| OP159446 | EPI_ISL_12983554 | 07-Mar-22 | Delta | XK00819-0819 |
| OP159581 | EPI_ISL_13094354 | 07-Mar-22 | Delta | XK00819-0821 |
| OP132702 | EPI_ISL_12982426 | 09-Mar-22 | Delta | XK00819-0826 |
| OP159447 | EPI_ISL_12983555 | 09-Mar-22 | Delta | XK00819-0827 |
| OP159409 | EPI_ISL_12983517 | 10-Mar-22 | Omicron | XK00819-0829 |
| OP159410 | EPI_ISL_12983518 | 10-Mar-22 | Delta | XK00819-0830 |
| OP159411 | EPI_ISL_12983519 | 10-Mar-22 | Delta | XK00819-0831 |
| OP159412 | EPI_ISL_12983520 | 10-Mar-22 | Delta | XK00819-0833 |
| OP159413 | EPI_ISL_12983521 | 11-Mar-22 | Omicron | XK00819-0834 |
| OP159414 | EPI_ISL_12983522 | 11-Mar-22 | Omicron | XK00819-0835 |
| OP159415 | EPI_ISL_12983523 | 14-Mar-22 | Omicron | XK00819-0836 |
| OP159416 | EPI_ISL_12983524 | 14-Mar-22 | Omicron | XK00819-0837 |
| OP159417 | EPI_ISL_12983525 | 14-Mar-22 | Omicron | XK00819-0839 |
| OQ028429 | EPI_ISL_15973673 | 15-Mar-22 | Omicron | XK00819-0841 |
| OQ028462 | EPI_ISL_16003857 | 15-Mar-22 | Omicron | XK00819-0842 |
| OP159418 | EPI_ISL_12983526 | 15-Mar-22 | Omicron | XK00819-0843 |
| OP159419 | EPI_ISL_12983527 | 15-Mar-22 | Omicron | XK00819-0844 |
| OP132713 | EPI_ISL_12987025 | 16-Mar-22 | Omicron | XK00819-0847 |
| OP132714 | EPI_ISL_12987026 | 16-Mar-22 | Omicron | XK00819-0849 |
| OP132715 | EPI_ISL_12987027 | 16-Mar-22 | Delta | XK00819-0850 |
| OP132716 | EPI_ISL_12987028 | 21-Mar-22 | Omicron | XK00819-0851 |
| OP132717 | EPI_ISL_12987029 | 21-Mar-22 | Omicron | XK00819-0852 |
| OP132718 | EPI_ISL_12987030 | 21-Mar-22 | Omicron | XK00819-0853 |
| OP132719 | EPI_ISL_12987031 | 21-Mar-22 | Omicron | XK00819-0854 |
| OP132720 | EPI_ISL_12987032 | 21-Mar-22 | Omicron | XK00819-0855 |
| OP132738 | EPI_ISL_12987050 | 22-Mar-22 | Omicron | XK00819-0856 |
| OP132739 | EPI_ISL_12987051 | 22-Mar-22 | Omicron | XK00819-0857 |
| OP132740 | EPI_ISL_12987052 | 22-Mar-22 | Omicron | XK00819-0858 |
| OP132741 | EPI_ISL_12987053 | 22-Mar-22 | Omicron | XK00819-0859 |
| OP132742 | EPI_ISL_12987054 | 22-Mar-22 | Delta | XK00819-0860 |
| OP132743 | EPI_ISL_12987055 | 23-Mar-22 | Omicron | XK00819-0861 |
| OP132744 | EPI_ISL_12987056 | 23-Mar-22 | Omicron | XK00819-0862 |
| OP132745 | EPI_ISL_12987057 | 23-Mar-22 | Omicron | XK00819-0863 |
| OP132746 | EPI_ISL_12987058 | 23-Mar-22 | Omicron | XK00819-0865 |
| OP132748 | EPI_ISL_12987060 | 24-Mar-22 | Omicron | XK00819-0870 |
| OP132749 | EPI_ISL_12987061 | 28-Mar-22 | Omicron | XK00819-0874 |
| OP132750 | EPI_ISL_12987062 | 28-Mar-22 | Omicron | XK00819-0875 |
| OP132751 | EPI_ISL_12987063 | 28-Mar-22 | Delta | XK00819-0876 |
| OP159486 | EPI_ISL_13091961 | 29-Mar-22 | Omicron | XK00819-0877 |
| OP159487 | EPI_ISL_13091962 | 29-Mar-22 | Omicron | XK00819-0879 |
| OP159488 | EPI_ISL_13091963 | 30-Mar-22 | Omicron | XK00819-0881 |
| OP159489 | EPI_ISL_13091964 | 30-Mar-22 | Omicron | XK00819-0883 |
| OQ028396 | EPI_ISL_15973640 | 30-Mar-22 | Omicron | XK00819-0884 |
| OP159490 | EPI_ISL_13091965 | 30-Mar-22 | Omicron | XK00819-0885 |
| OP159491 | EPI_ISL_13091966 | 31-Mar-22 | Omicron | XK00819-0887 |
| OP159492 | EPI_ISL_13091967 | 31-Mar-22 | Omicron | XK00819-0888 |
| OP159493 | EPI_ISL_13091968 | 01-Apr-22 | Omicron | XK00819-0894 |
| OP159494 | EPI_ISL_13091969 | 01-Apr-22 | Omicron | XK00819-0895 |
| OP159495 | EPI_ISL_13091970 | 04-Apr-22 | Omicron | XK00819-0899 |
| OQ028399 | EPI_ISL_15973643 | 04-Apr-22 | Omicron | XK00819-0900 |
| OQ028403 | EPI_ISL_15973647 | 07-Apr-22 | Omicron | XK00819-0910 |
| OP159508 | EPI_ISL_13091983 | 07-Apr-22 | Omicron | XK00819-0913 |
| OP159509 | EPI_ISL_13091984 | 07-Apr-22 | Omicron | XK00819-0914 |
| OQ028404 | EPI_ISL_15973648 | 08-Apr-22 | Omicron | XK00819-0915 |
| OQ028419 | EPI_ISL_15973663 | 18-Apr-22 | Omicron | XK00819-0920 |
| OQ028420 | EPI_ISL_15973664 | 18-Apr-22 | Omicron | XK00819-0921 |
